# Quantification of Epstein-Barr Virus DNA in Patients with Idiopathic Orbital Inflammatory Pseudotumor

**DOI:** 10.1371/journal.pone.0050812

**Published:** 2013-01-02

**Authors:** Rui Jin, Pengxiang Zhao, Xuemei Ma, Jianmin Ma, Yanan Wu, Xinji Yang, Jingxue Zhang, Rugang Zhong, Yi Zeng

**Affiliations:** 1 College of Life Science and Bio-engineering, Beijing University of Technology, Beijing, People's Republic of China; 2 Beijing Ophthalmology and Vision Science Key Lab, Beijing Tongren Eye center, Beijing Tongren Hospital, Capital Medical University, Beijing, People's Republic of China; 3 Institute of Orbital disease, Chinese Armed Police General Hospital, Beijing, People's Republic of China; 4 Chinese Center for Disease Control and Prevention, Beijing, People's Republic of China; Nanyang Technological University, Singapore

## Abstract

Inflammatory pseudotumors (IPT) are soft tissue tumors that include a diverse group of lesions characterized by inflammatory cell infiltration and variable fibrotic responses. Idiopathic orbital inflammatory pseudotumors (IOIP) are IPTs of unknown etiology that develop in the orbit. Due to the lack of well-defined pathogenic mechanisms, diagnosis and treatment of this disease remain a significant challenge. Epstein-Barr virus (EBV) infection, which causes significant lymphocyte infiltration, has been proposed to be involved in IOIP. This study tries to validate the relationship between EBV infection and the development of IOIP. Sixteen IOIP tissue samples were obtained from patients during surgical resection of the lesion. One Graves' ophthalmopathy tissue sample and 20 normal donors' plasma serves as controls. The plasma level of five EBV antibodies, including VCA-IgG, VCA-IgA, VCA-IgM, EA-IgG and EBNA1-IgG were examined. All plasma samples were EB-VCA-IgG positive and EB-VCA-IgM negative, suggesting that all people tested had been infected with EBV but not in the acute infection stage. EBV-DNA was detected in 15/16 (94%) of IOIP tissue samples despite different levels of lymphocyte infiltration and 5/16 plasma samples (31%) were detected EBV DNA positive which is higher than the normal controls (10%). Percent of positive plus suspected positive samples with one or more of the three important risk markers (VCA-IgA, EA-IgG, EBV-DNA) is 50% of the patients (8/16) which is much higher compare with the normal controls (20%). The results further reveal the relationship between IOIP and EBV infection.

## Introduction

Inflammatory pseudotumors (IPT), also known as inflammatory myofibroblastic tumors (IMT), are soft tissue tumors that include a diverse group of lesions characterized by inflammatory cell infiltration and variable fibrotic responses [1]. This type of tumor contains differentiated myofibroblastic spindle cells, lymphocytes, and eosinophils [2]–[5]. It may occur in many organs including liver [6], lung [7], bladder [8], spleen [9], lymph nodes [10], kidney [11] and eye [12] in both children and adults, with possible systemic symptoms, occasional recurrence, and rare malignant transformation.

Idiopathic orbital inflammatory pseudotumors (IOIP) are one of the most common IPT diseases of the orbit, which can not only affect one's appearance due to proptosis and eyelid or conjunctival congestion, but also impair vision. After Graves' disease and lymphoproliferative disorders, IOIP is the 3rd most common ophthalmologic disease of the orbit and account for approximately 7–12.3% of all the orbital tumors [13], [14]. Currently, diagnosis relies on histological examination or immunologic staining of the lesion obtained by gross resection or biopsy. Surgical resection is the treatment of choice for most cases, corticosteroids and other anti-inflammatory drugs can also be used, but associated with a higher incidence of relapse. As the etiology of IOIP is not clear, diagnosis and treatment remains a significant challenge in ophthalmology.

Although various etiologies of IOIP have been proposed pertaining to the origin of the lesion, *viz*, minor trauma, surgery and the origin of autoimmunity, the exact pathophysiology of the disease remains unknown. IOIP has been shown to be associated with the occurrence of upper respiratory tract infection, viral influenza, syphilis and paranasal sinusitis [15]–[17].

EBV is a common herpesvirus that usually infects lymphocytes and epithelial cells and was specifically detected in the tumor by in situ hybridization for EBERs in lymph nodes-derived IPT, splenic IPT, liver IPT and spleen IPT [18], [19]. In our former study [20], we have identified 90% (9/10) EBV positive IOIPs using PCR, and all four control samples were negative. Also, the high level expression of EBI2 in the IOIP group indicating that EBV infection may be related to the development of IOIP. EBV is reported capable of infecting B lymphocytes, squamous epithelial cells, glandular epithelial cells, myoepithelial cells, smooth muscle cells, T cells and NK cells, etc [21]. Epstein-Barr virus-induced receptor 2 (EBI2) is an orphan seven-transmembrane (7TM) receptor originally identified as the most up-regulated gene (>200-fold) in EBV-infected cells [22]. EBI2 is expressed in B-lymphocyte cell lines and in lymphoid tissues; the up-regulation of EBI2 and the presence of EBV-DNA in IOIPs may due to the high prevalence of EBV infection in population and the lymphocytes infiltration in IOIPs.

In an effort to shed light on the role of EBV in the IOIP pathogenesis, EBV-DNA, EB-VCA-IgG, VCA-IgA, VCA-IgM, EA-IgG and EBNA1-IgG were used to evaluate EBV infection in IOIP patients respectively.

## Results

### A. Serologic Test for EBV Antibodies

The antibody titers of EBV, including VCA-IgG, VCA-IgA, VCA-IgM, EA-IgG and EBNA1-IgG, were examined in the above 16 IOIP patients' and 20 normal donors' plasma which are served as the control (Table 1).

**Table 1:** Results of EBV antibodies and DNA detection in plasma of IOIP patients and healthy controls.

Detecting items	Normal (n = 20)		Positive rate (%)	IOIP (n = 16)		Positive Rate (%)
	Positive	Suspected		Positive	Suspected	
VCA-IgG	20	0	100	16	0	100
VCA-IgM	0	0	0	0	0	0
EBNA1-IgG	18	0	90	15	0	94
VCA-IgA	1	0	5	3	1	19 (6*)
EA-IgG	1	0	5	4	1	25 (6*)
EBV-DNA	2	-	10	5	-	31
Total**	-	-	20	7	1	44(6*)

* Percent of suspected positive samples.

** Percent of positive samples with one or two of the three important risk markers (VCA-IgA, EA-IgA, EBV-DNA) positive or suspected positive.

Detection of these five kinds of antibodies may tell the stage of EBV infection of the seven IOIP patients. About two weeks after infection, anti-EA IgG, anti-VCA IgM and anti-VCA IgG-antibodies can be detected in the patient's serum. EBV-VCA IgM is produced in all primary EBV infections, whether symptomatic or asymptomatic. If antibody to EBNA is positive along with VCA-IgG, a diagnosis of a primary infection can be excluded. If the EBNA titer is negative in the presence of VCA IgG and IgM, a primary infection is likely. In this study, all IOIPs are seropositive of EB-VCA-IgG, fifteen of sixteen are EBNA1-IgG positive (93.8%), but none are VCA-IgM positive. Compared to the IOIPs, the normal donors' has a positive rate of 100%, 0%, 5%, 10%, and 90% in the detection of EBV-VCA-IgG, IgM, IgA, EA-IgG, and EBNA1-IgG. These suggest that all the sixteen IOIP patients and twenty normal donors had previous EBV infection. In most cases of reactivations, e.g. in which caused by immunosuppression, the anti-EA IgG and anti-VCA IgA antibody concentration increase. Therefore positive reaction of anti-EA-IgG and anti-VCA IgA is a noticeable marker for reactivations. In these studies, two patients are positive of both VCA-IgA and EA-IgG, one is suspected positive of both VCA-IgA and EA-IgG, one is positive of VCA-IgA and two are positive of EA-IgG (Table 1&2). The percent of positive plus suspected positive samples with at least one antibody (VCA-IgA, EA-IgG) is 38% (6/16).

**Table 2:** Results of EBV VCA-IgA, EA-IgG and DNA detection of Group T patients.

samples	EBV DNA		EBV Antibody		Histopathology (figure 1)
	Tissue	Plasma	VCA-IgA	EA-IgG	
A	+	−	−	+	dense fibrous connective with scattered lymphocytes infiltration
B	+	+	+	+	
C	+	−	−	−	
D	+	−	+	−	dense polymorphous lymphocytes infiltration with little fibrosis
E	+	−	+	+	
F	+	+	−	+	
G	+	+	−	−	wide glandular epithelial cells with a few lymphocytes infiltration
H	+	−	−	−	
I	+	−	−	−	

### B. EBV-DNA Detection

In our former study [20], EBV-DNA was detected in 9/10 (90%) of the IOIP tissue samples. In this study, One Graves' ophthalmopathy tissue sample with obvious inflammatory cell infiltration was chosen as the control. EBV DNA was detected in 15/16 of IOIP patients' tissue samples (94%) but not in the control's tissue sample.

We examined plasma EBV DNA in the above sixteen IOIPs and twenty normal donors which served as the control. Five of sixteen IOIP patients showed EBV DNA positive (31%) while only two of twenty (10%) in normal controls (Table 1 & 2). All of the EBV positive IOIP serology patients also positive for EBV DNA in their tumor tissue.

**Figure 1:**
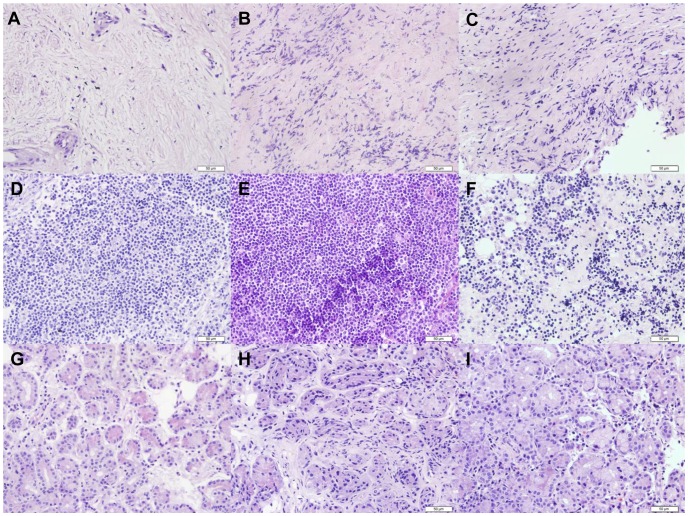
Histological analysis of the tissue paraffin section of Group T patients. Tissues of Group T patients are of different levels of lymphocyte infiltration. A, B, and C are the fibrosis type, represent dense fibrous connective with scattered lymphocytes infiltration; D, E, and F are the lymphocyte infiltration type with dense polymorphous lymphocytes infiltration; G, H, I mainly consist of glandular epithelium cells with a few lymphocytes infiltration. H&E stain, original magnification 40×.

EBV-DNA in IOIP tissues might originate from different kind of cells.To further analysis if the EBV DNA originate from inflammatory cells in the tissues only, nine of the sixteen tissue section (group T patients) were stained with hematoxylin and eosin (H & E). Histological pictures showed that tissues of Group T patients are of different types and have different levels of lymphocyte infiltration (table 2, figure 1). Of the nine cases, EBV DNA has a high level of expression in all of the tissue samples and in 3 of the 9 plasma samples. Of the three plasma EBV-DNA positive cases, One is both VCA-IgA and EA-IgG positive, one is VCA-IgA positive, one is only positive in plasma EBV-DNA. It is suggested that the high positive incidence of EBV-DNA detection in IOIP tissues might be not only the results of the lymphocytes infiltration in inflammation response.

These results might not support the possibilities that EBV-DNA positive in IOIP tissues is only attributable to the presence of inflammatory cells in the tissues. It is indicated that EBV infection was not fortuitous and EBV may be an important factor in the etiopathology of IOIP.

## Discussion

Despite of the high prevalence of EBV infection in population and the lymphocytes infiltration in IOIP tissues, it is still possible that EBV infection may be associated with IOIP etiopathologensis.

### C. EBV infection is associated with IOIP pathogenesis

Several groups have reported that EBV RNA (EBERs) can be detected in lymph nodes-derived IPT, splenic IPT and liver IPT by *in situ* hybridization. A subset of IPTs, particularly those in the spleen and liver, has been shown to harbor EBV in spindle cells [18], [19].

In this study, all sixteen IOIP patients and the normal controls are EBV seropositive persons, and have been infected with EBV. EBV DNA were detected in fifteen of sixteen IOIP patients tissue samples (94%) despite different levels of lymphocyte infiltration and in five of sixteen plasma samples (31%), but not in the control Graves' ophthalmopathy tissue with the infiltration of inflammatory cells and only in two of twenty (10%) normal plasma controls. The results further reveal the relationship between IOIP and EBV infection. It is suggested that the high positive incidence of EBV-DNA detection in IOIP tissues is not only the results of the lymphocytes infiltration in inflammation response.

### D. IOIP and EBV-associateed diseases

EBV has been found in tissue of nasopharyngeal carcinoma (NPC) [23], sinnasal undifferentiated carcinoma, lymphomas, stomach, lung and thymus.

Since IOIP is related to EBV infection, it might have relationship with other EBV-associated diseases, especially NPC. About 13.1–29% of NPC patient are associated with eye symptoms [24]. Many NPC patients with eye symptoms usually go to see the oculist first and are occasionally misdiagnosed with ocular diseases. Currently, the serum immunoglobulin A against Epstein-Barr virus capsid antigen (VCA-IgA) is one of the most commonly used markers for diagnosis of NPC [25]. Lin investigated the clinical significance of plasma concentrations of EBV DNA in patients with NPC and found that plasma EBV DNA was detectable before treatment in 94 of the 99 patients, but not in 40 healthy controls or 20 cured patient [26]. In this study, two patients are positive of VCA-IgA and EA-IgG, one is suspected positive of VCA-IgA and EA-IgG, one is positive of VCA-IgA and two are positive of EA-IgG (Table 1&2), five patients were higher in plasma EBV-DNA level, represent 50% of the patients (8/16). While only 20% (4/20) of the normal donors were detected positive in the three risk markers (VCA-IgA, EA-IgG, EBV-DNA, Table 1). The IOIP patients might be the high risk group of NPC or at the early stage of EBV re-activation stage and need to keep a close watch on the EBV infection status.

Eye is very sensitive, any pathological changes appear in orbital cavity is easier to find than in other tissues. The intensive study of the relationship between the IOIPs and NPC or other EBV related diseases might provide an early warning for these kinds of diseases, but its essence still needs to be validated. Also, for lacking of animal model, we know very little about the re-activation and carcinogenic mechanism of EBV. IOIP patients might be at the very early stage of EBV re-activation and could serve as a good model for the study of EBV pathogenesis.

In conclusion, our results further demonstrated the relationship between IOIP and EBV infection. Further investigations might be of great value in better understanding the relationship of IOIP and EBV infection.

## Methods

### E. Ethics Statement

The study “Idiopathic Orbital Inflammatory Pseudotumor” was approved by the Institutional Review Board of the Institute of Orbital Diseases, and written informed consent was provided by all study participants and/or their legal guardians.

### F. Clinical Samples

Sixteen IOIP tissue samples and one Graves' ophthalmopathy tissue sample were obtained from patients during surgical resection of the lesion (IOIPs) at Beijing Armed Police General Hospital between 2009 and 2012. Twenty normal donors'plasma samples were provided by Beijing Tongren Hospital in 2011. Nine of the sixteen tissue sections (group T patients) were stained with hematoxylin and eosin (H & E). Three of the tissue sections have large number of lymphocyte infiltration, three has middle to a few lymphocyte infiltration with dense fibrous connective, three has only very few lymphocyte infiltration with wide glandular epithelium cells. The Graves' ophthalmopathy tissue sample with obvious inflammatory cell infiltration was chosen as the control. The tissue samples were immediately snap-frozen and stored in liquid nitrogen and plasma samples were stored in −80°C refrigerator. The IOIP diagnosis was made based on pathological examinations of hematoxylin-eosin-stained tissue sections.

### G. DNA Extraction from Samples

DNA was purified from tissue samples and plasma samples using the DNeasy Mini Kit (Qiagen, Valencia, CA, USA) according to the manufacturer's instructions. The quality and the quantity of the DNA were assessed by agarose gel electrophoresis and spectrophotometrical analysis.

### H. QPCR test of EBV-DNA

EBV DNA was amplified using the EBV-DNA detection kit purchased from DaAn Gene Co. Ltd. (licensed by China State Food and Drug Administration) and crude DNA as the template in a 50 µl reaction. Nuclease-free distilled water was used as a negative control to ensure that the reagents used were not contaminated. The positive DNA control and primers for EBV were supplied with the EBV-DNA detection kit. Real-time quantitative PCR is based on the continuous optical monitoring of the progress of a fluorogenic PCR reaction. In this system, one fluorescent dye serves as a reporter (FAM), and its emission spectra is quenched by a second fluorescent dye (TAMRA). During the extension phase of PCR, the 5′ to 3′ exonuclease activity of Taq DNA polymerase cleaves the reporter from the probe, thus releasing it from the quencher and resulting in an increase in fluorescence emission at 518 nm. The reactions were subjected to 30 cycles of amplification: denaturation at 95°C for 45 sec, annealing of primers at 55°C for 45 sec, and extension of the primers at 72°C for 45 sec. Fluorescence was detected at the end of the 72°C segment in the PCR step. The results were analysed by using MxPro™ QPCR Software.

### I. Enzyme-linked Immunosorbent Assay for plasma

EB-VCA-IgG, VCA-IgA, VCA-IgM, EA-IgG and EBNA1-IgG were analyzed with enzyme-linked immunosorbent assay (ELISA) kits (EUROIMMUN Corporation, Germany). A 96-well microplate was prepared and coated with capture antibody. Samples or standards were added, then the plates were washed, horseradish peroxidase labeled detection antibody was added to each well, and washing was repeated three times. Finally, stop solution was added to each well. The optical densities of each well were quantified in 30 minutes at dual wavelengths of 450 nm using a micro-plate reader (PerkinElmer, USA).
